# Toxic Prediction of Pyrrolizidine Alkaloids and Structure-Dependent Induction of Apoptosis in HepaRG Cells

**DOI:** 10.1155/2021/8822304

**Published:** 2021-01-02

**Authors:** Pimiao Zheng, Yuliang Xu, Zhenhui Ren, Zile Wang, Sihan Wang, Jincheng Xiong, Huixia Zhang, Haiyang Jiang

**Affiliations:** Department of Pharmacology and Toxicology of the College of Veterinary Medicine, China Agricultural University, Beijing Key Laboratory of Detection Technology for Animal-Derived Food Safety, Beijing Laboratory for Food Quality and Safety, Beijing 100193, China

## Abstract

Pyrrolizidine alkaloids (PAs) are common phytotoxins and could cause liver genotoxicity/carcinogenicity following metabolic activation. However, the toxicity of different structures remains unclear due to the wide variety of PAs. In this study, the absorption, distribution, metabolism, excretion, and toxicity (ADMET) of 40 PAs were analyzed, and their toxicity was predicted by Komputer Assisted Technology (TOPKAT) using Discovery Studio software. The in silico results showed that all PAs except retronecine had good intestinal absorption, and all PAs were predicted to have different toxicity ranges. To verify the predictive results, 4 PAs were selected to investigate cell injury and possible mechanisms of the differentiation in HepaRG cells, including retronecine type of twelve-membered cyclic diester (retrorsine), eleven-membered cyclic diester (monocrotaline), noncyclic diester (retronecine), and platynecine type (platyphylline). After 24 h exposure, retronecine-type PAs exhibited concentration-dependent cytotoxicity. The high-content screening assay showed that cell oxidative stress, mitochondrial damage, endoplasmic reticulum stress, and the concentration of calcium ions increased, and neutral lipid metabolism was changed notably in HepaRG cells. Induced apoptosis by PAs was indicated by cell cycle arrest in the G2/M phase, disrupting the mitochondrial membrane potential. Overall, our study revealed structure-dependent cytotoxicity and apoptosis after PA exposure, suggesting that the prediction results of in silico have certain reference values for compound toxicity. A 1,2-membered cyclic diester seems to be a more potent apoptosis inducer than other PAs.

## 1. Introduction

Pyrrolizidine alkaloids (PAs), a group of secondary plant metabolites, belong to the most widely distributed natural toxins [1]. Intake of PAs present in plants and/or contaminated foodstuffs causes numerous cases of toxicity [2, 3]. Tea, honey, and herbal spices were identified as the primary sources contributing to human exposure to PAs [1, 4, 5]. Different doses of PAs could cause acute toxicity and chronic toxicity [6]. Acute intoxication with high doses of PAs in humans is characterized by hepatomegaly and ascites, accompanied by high mortality rates [7].

PAs represent a diverse class of heterocyclic alkaloids with a necine base and a necic acid, according to the structure of the necine base [8]. PAs are classified into three types: retronecine type (including its 7-stereoisomer), otonecine type, and platynecine type. The former two kinds of PAs possess a 1,2-double bond in the necine base as the unsaturated PAs, whereas platynecine-type PAs as the saturated PAs because of the missing double bond. Furthermore, they could be grouped according to their degree of esterification into monoesters, noncyclic diesters, and cyclic diesters [9, 10]. Due to different combinations of necine bases and necine acid, there are a wide variety of PA structures. Thus, a comprehensive assessment of the relationship between structures and toxicity of PAs is very important for food safety.

The current toxicity studies on PAs are mainly based on animal experiments [11–13]. For a wide variety of PAs, the use of large number experimental animals for toxicological assessment does not meet the principles of animal welfare, and the test cycle is long, costly, and inefficient. The network toxicology model used for toxicity prediction is convenient and fast, significantly improving toxicity assessment efficiency [14]. Evaluating the body involving absorption, distribution, metabolism, excretion, and toxicity (ADMET) properties are essential for compound performance [15]. Current knowledge of ADMET and TOPKAT is primarily built on the chemical structure and the physicochemical properties determined by its structure [16–18].

HepaRG cells possess a hepatocyte-like morphology, maintaining hepatic functions and expression of liver-specific genes, including the expression of critical metabolism enzymes, drug transporters, and nuclear receptors, such as CYP enzymes, phase II enzymes, albumin, transferrin, aldolase B, and apical and canalicular ABC transporters and nuclear receptors (CAR, PXR) at levels comparable to human primary hepatocytes [19, 20]. Due to these properties, the HepaRG cell line, with its metabolic competence and long-term cultivability, is a beneficial *in vitro* test system to assess the hepatocellular toxicity induced by plant extracts [21, 22].

In this study, 40 PAs were predicted potential ADMET and TOPKAT using the Discovery Studio software. To verify the prediction results, 4 PAs were selected to investigate cell injury and possible mechanisms of differentiation in HepaRG cells, including retronecine type of twelve-membered cyclic diester (retrorsine), eleven-membered cyclic (monocrotaline) and noncyclic (retronecine) diester, and platynecine type (platyphylline). Then, the differentiation HepaRG hepatocyte cells were used to analyze the *in vitro* cytotoxic. The high-content screening (HCS) assays were conducted based on five parameters (oxidative stress, mitochondrial damage, endoplasmic reticulum dysfunction, disorders of neutral lipid metabolism, and calcium homeostasis). In addition, cell cycle, mitochondrial membrane potential, and apoptosis were all analyzed to identify the effects of PAs with different structures on toxicity.

## 2. Materials and Methods

### 2.1. Chemicals

Retrorsine (RTS), monocrotaline (MCT), retronecine (RN), platyphylline (PLA), and camptothecin (CPT) were purchased from J&K Scientific Ltd. (Beijing, China). 0.25% Trypsin/1 mM EDTA, 0.25% Trypsin, William's E Medium (no phenol red), Dulbecco's phosphate-buffered saline (DPBS), fetal bovine serum (FBS, Australia Origin), Dead Cell Apoptosis Kit with Annexin V Alexa Fluor™ 488 and Propidium Iodide (PI), Tali™ Cell Cycle Kit, the fluorescent probes Hoechst 33342, Mito Tracker Red CMXRos, ER-Tracker™ Green (BODIPY™ FL Glibenclamide) for live-cell imaging, BODIPY™, Fluo-4 direct calcium reagent, Total Reactive Oxygen Species (ROS) Assay Kit 520 nm, and Nunclon™ Sphera™ Microplates (96 U-Well Plate) were purchased from Thermo Fisher Scientific (Waltham, MA, USA). The Cell Counting Kit-8 and Mitochondrial Membrane Potential Assay Kit with JC-1 were purchased from Beyotime (Nanjing, China). Hydrocortisone-hemisuccinate (sodium salt) and dimethyl sulfoxide (DMSO) were acquired from Solarbio Science﹠Technology Co., Ltd. (Beijing, China). The HepaRG cell line was purchased from Millipore (BeNa Culture Collection, China).

### 2.2. ADMET and TOPKAT Analyses

ADMET analysis and toxicity profiling (TOPKAT) of PAs were performed using Discovery Studio 2019 (Accelrys, Inc., San Diego, CA, USA). The ADMET analysis included aqueous solubility (AS), blood–brain barrier (BBB), cytochrome P450 2D6 (CYP2D6), plasma protein binding (PPB), and hepatotoxicity (HT) descriptors. The toxicity prediction profile included rodent carcinogenicity (based on the U.S. National Toxicology Program dataset), developmental toxicity potential properties, Ames mutagenicity, and rat oral LD_50_ and chronic oral LOAEL.

### 2.3. Cell Culture

The human hepatic cell line HepaRG was cultured in a growth medium consisting of William's Medium E (10% fetal bovine serum, 1% 100 U/mL penicillin, 1% 100 *μ*g/mL streptomycin, and 50 *μ*M hydrocortisone hemisuccinate), then cultured in a cell incubator at 37°C and 5% CO_2_. For toxicity studies (cell viability and PA induction studies), HepaRG cells were seeded in Nunclon™ Sphera™ Microplates (Thermo Fisher Scientific; 9000 cells per well in 100 *μ*L). The process of cell differentiation is described previous literature [23]. Before toxicity studies, differentiated HepaRG cells were incubated in assay medium (growth medium containing 2% FBS) supplemented with 0.5% DMSO. For all assays, PAs were dissolved in PBS (0.05 M, pH 7.4) to make a stock solution of 100 mM. At this stage, the cells were ready to be used for toxicity studies. Cells that were not immediately used were kept in a differentiation medium for a maximum of three additional weeks. The medium was refreshed every 1-2 days during culturing.

### 2.4. Cell Viability Assay

The effect of PAs on cell viability was determined using the Cell Counting Kit-8 (CCK-8) assay [24]. After differentiation, HepaRG cells were seeded in 96-well plates at a density of 1 × 10^3^~10^4^ cells/well. The cells were incubated with different concentrations (0, 6.25, 12.5, 25, 50, 100, 200, 400, and 800 *μ*M) of RTS, MCT, RN, and PLA for 24 h. CPT (10 *μ*M) was used as a positive control for cytotoxicity. After the respective incubation period, 10 *μ*L of undiluted CCK-8 reagent were added to each well and incubated in the dark for 2 h at 37°C. Subsequently, the absorbance at 450 nm was detected with a microplate reader (Thermo Scientific, Waltham, MA, USA). The cell viability rate was calculated using the following formula:

(1) $$\begin{array}{c} \text{viability rate}\left(\%\right)=\left({\text{OD}}_{\text{sample}}-{\text{OD}}_{\text{vehicle}}\right)/\left({\text{OD}}_{\text{control}}–{\text{OD}}_{\text{vehicle}}\right)\times 100\%. \end{array}$$

### 2.5. High-Content Screening Assay

Cells were seeded in a 96-well microplate (3 ~ 5 × 10^3^ cells/well) and treated with RTS, MCT, RN, and PLA (0, 50, 200, 400, and 800 *μ*M) for 24 h. 10 *μ*M CPT was used as a positive control. There are 6 dyes prepared as fluorescence probes and marked with cell number (Hoechst 33342), mitochondrial membrane potential (Mito Tracker), endoplasmic reticulum (ER-Tracker), reactive oxygen species (BODIPY), calcium ions (Fluo-4), neutral lipids, oils, and polymers (DCFH-DA). Dyes were excited, and their fluorescence was monitored at the excitation and emission wavelengths with appropriate filter settings [25]. Considering the overlapping emission and absorption spectra of different fluorescent probes, we prepared four strategies: Hoechst 33342 was combined with Mito Tracker/DCFH-DA, ER-Tracker, BODIPY, and Fluo-4, respectively. After 0.5 h of incubation, cells were washed three times with DPBS. The cells were then imaged and analyzed using the ImageXpress and MetaXpress High Content Imaging System (Molecular Devices Corporation, USA). Nine randomized images of each culture well were acquired successively with an ×20 objective with at least 500 cells collected in each well. The parameters of subcellular structures, which were stained by different fluorescent probes, were quantified using High Content Image Acquisition and Analysis Software Version 6.0. The average fluorescence intensity was acquired for further analysis.

### 2.6. Cell Cycle Assay

1 × 10^6^ cells/well HepaRG cells were seeded in 6-well plates and were treated with RTS, MCT, RN, and PLA at concentrations of 0, 50, and 200 *μ*M for 24 h. CPT was used as a positive control. After the incubation period, the HepaRG cells were digested by a 0.25% trypsin solution (without EDTA) for 5~10 min at 37°C, resuspended in culture medium, centrifuged for 1000 rpm 3 min, washed in DPBS, and were collected. The collected cells were fixed with 70% ice-cold ethanol at 4°C kept overnight and stained with PI/RNase (staining buffer solution) for 30 min at 37°C in the dark. The distribution of the cells in each stage and the DNA content of cells were analyzed by BD FACSVerse flow cytometer (BD Biosciences, Heidelberg, Germany) [22].

### 2.7. Cellular Apoptosis Assay

The induction of apoptosis or necrosis was assayed by using the Apoptosis Kit with Annexin V Alexa Fluor™ 488 & Propidium Iodide (PI) [26]. HepaRG cells were seeded, treated, and collected as described in Cell Cycle Assay. According to the instructions of the kit (Thermo Fisher Scientific), the dye to the required concentration was prepared. 100 *μ*L of cell suspension (10^6^ cells/mL) were either resuspended in 195 *μ*L of 1× Annexin V binding buffer and then incubated with 5 *μ*L of Annexin V-FITC and 10 *μ*L of PI in the dark for 15 min at room temperature (RT). Then, the cells were quickly subjected to flow cytometry. The percentage of apoptotic cells was calculated.

### 2.8. Mitochondrial Membrane Potential JC-1 Assay

The mitochondrial transmembrane potential was evaluated using JC-1, a mitochondria-specific lipophilic cationic fluorescence dye [23]. HepaRG cells were seeded, treated, and collected as described in Cell Cycle Assay. The cells were then incubated with 1X JC-1 dye for 20 min at 37°C in the dark and washed twice with the dye buffer. Finally, the cells were quickly subjected to flow cytometry.

### 2.9. Statistical Analysis

Each experiment was repeated at least thrice independently. Data were analyzed using SPSS and are shown as the mean ± SD. To evaluate a statistical difference between the treatments and the solvent control. one-way ANOVA with *p* < 0.05 was considered significant.

## 3. Results and Discussion

### 3.1. ADMET and TOPKAT Assays

Current knowledge of in silico ADMET and TOPKAT are primarily built on the drug's chemical structure and physicochemical properties [16]. The information helps reduce the time and computational cost of screening compound libraries to select feasible compounds for synthesis and further testing [27]. The results are shown in Table 1. Based on the ADMET findings, all types of PAs except RN showed good intestinal absorption, good aqueous solubility, low to undefined blood–brain barrier (BBB) penetration, and not the inhibitor of CYP2D6. Both retronecine type and otonecine type showed a hepatotoxicity effect. All platynecine-type PAs except PLA showed nontoxic, which is not consistent with the performance of the literature reported [9]. It may be that PLA produces substances in the metabolic process, which will cause toxicity at high concentrations [28, 29]. For protein plasma binding (PBB), except PLA, all PAs were predicted to be highly bound to protein plasma.

TOPKAT is a common tool for predicting the potential ecotoxicity, toxicity, mutagenicity, and reproductive or developmental toxicity of drug candidates [30]. The 40 PAs were screened with TOPKAT for the following toxicity prediction properties: rodent carcinogenicity, Ames mutagenicity, and developmental toxicity potential properties, as well as rat oral LD_50_ and chronic oral LOAEL (Table 2). According to the two models-NTP ccinogenicity call, all compounds have different degrees of carcinogenicity. In the mutagenicity predictor (Ames heteroaromatics model), a few compounds of PAs were found to be mutagenic. All PAs are potentially dangerous against developmental toxicity. Moreover, relatively low LD_50_ and low chronic oral LOAEL *in vivo* were predicted for these 40 compounds. As shown in Tables 1 and 2, the hepatotoxicity of PA rank order for necine base is otonecine type > retronecine type > platynecine type, which consistent with the results of Schöning et al. [28]. With retronecine-type PAs tested, the 12-membered macrocyclic diesters exerted higher cytotoxicity than the monoester. This result was consistent with previous cytotoxic studies to investigate the relative cytotoxic potency of different PAs [31–33]. However, for 11-membered macrocyclic diesters, 12-membered macrocyclic diesters, and no-ester retronecine-type PAs, few studies compare their hepatotoxicity, especially retronecine. Because of its 1,2-saturated structure, PLA was considered nontoxic in the previous literature [9], but the prediction in silico result is hepatotoxicity.

**Figure 1:**
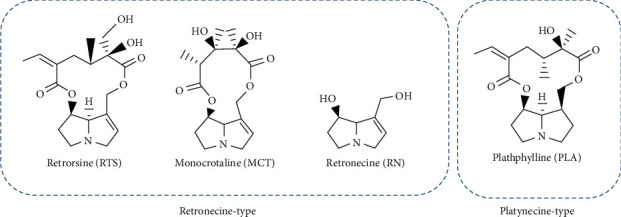
Chemical structures of the investigated PAs. Two classic structural types of PAs, which cover 4 different structures: retronecine type of twelve-membered cyclic diester (RTS), eleven-membered cyclic diester (MCT), noncyclic diester (RN), and platynecine-type (PLA).

Finally, we chose retronecine-type twelve-membered cyclic diester (retrorsine), eleven-membered cyclic diester (monocrotaline), noncyclic diester (retronecine), and platynecine type (platyphylline) for *in vitro* cytotoxic assay to verify the in silico prediction results (Figure 1).

### 3.2. Cell Viability Assay

**Figure 2:**
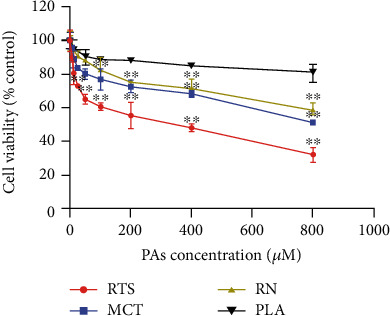
PA-induced cytotoxicity in HepaRG cells after 24 h exposure. Cells were seeded in 96-well plates and after differentiation and incubated with increasing concentrations (0, 6.25, 12.5, 25, 50, 100, 200, 400, and 800 *μ*M) of 4 PAs for 24 h. 10 *μ*M CPT was used as a positive control (data not shown). Data were normalized to solvent control and are shown as means ± SD of three independent experiments performed with five replicates each. (^∗∗^*p* < 0.05 one-way ANOVA followed by Dunnett's *t*-test).

The CCK-8 assay investigated the influence of four structurally different PAs on the viability of HepaRG cells after PA exposure in concentrations ranging between 6.25 *μ*M and 800 *μ*M for 24 h, to determine the appropriate concentrations for investigating a possible apoptotic or necrotic potential of PA. The *in vitro* metabolized 4 PAs led to a significant and concentration-dependent decrease in HepaRG viability (Figure 2). The results showed that RTS, MCT, and RN were cytotoxic, with a significant decrease in viability to 32.27%, 51.49%, and 59.39% for the highest exposure (800 *μ*M) as for PLA was 80.76%. Compared with platynecine type, retronecine type has more cytotoxic potency, and the order was RTS ≥ MCT ≥ RN ≥ PLA. The results demonstrated that the HepaRG cell model successfully discriminated against the cytotoxic potency of different PAs [31, 34].

### 3.3. High-Content Screening Assay

**Figure 3:**
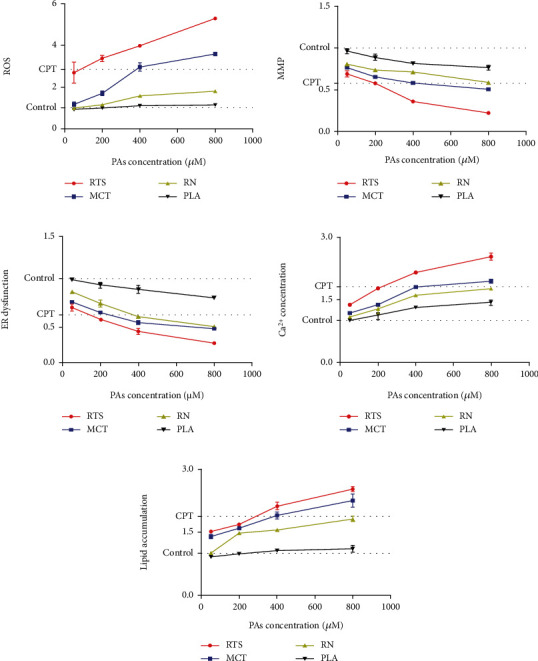
Representative data from the HCS assay in which the ROS (a), MMP (b), ER dysfunction (c), Ca^2+^ concentration(d), and lipid accumulation(e) in HepaRG cells treated with different concentrations (0, 50, 100, 200, 400, and 800 *μ*M) of 4 PAs for 24 h. After drug exposure, cell viability, oxidative stress, mitochondrial damage, endoplasmic reticulum dysfunction, disorders of neutral lipid metabolism, and calcium homeostasis were measured. All the parameters are expressed as the fold change compared to the control group. The data are presented as the mean of three replicate wells.

Different concentrations (0, 50, 200, 400, and 800 *μ*M) were selected to be incubated with HepaRG cells for 24 hours to explore the cytotoxicity of different structures and concentrations of 4 PAs. Camptothecin was used as a positive control for apoptosis [23]. The parameters studied in HCS were reactive oxidative stress (ROS), mitochondrial damage, endoplasmic reticulum dysfunction, disorders of neutral lipid metabolism, and calcium homeostasis followed by cytotoxic effects. Representative data was shown in Figure 3. The results showed that all 4 PAs could cause five parameters change in HepaRG cells with obvious dose dependence. Mainly, the data showed the increased reactive oxygen species (ROS) (0.93-5.30-fold), decreased mitochondrial membrane potential (MMP) (0.21-0.98-fold), accumulation of neutral lipids (0.88-2.60-fold), increased calcium ion concentration (0.99-2.61-fold), and endoplasmic reticulum dysfunction (0.21-1.00-fold) of the control vehicle in the HepaRG cell line. At the same dosage, the four alkaloids with different structures show apparent differences in toxicity. Among them, platynecine-type PLA did not show obvious cytotoxicity, which was consistent with the Discovery Studio software results, implying that the biological toxicity and structure of PAs had a more significant correlation. It is worth noting that among the various parameters measured by HCS, the change of ROS is the most obvious, and it is always accompanied by the decrease of MMP. This result suggests that cell injury induced by PAs may be mainly through oxidative stress. The toxic effects of PAs are considered cumulative [8]. If PAs were exposed to cells, it may cause cellular oxidative stress, leading to the accumulation of intracellular ROS. Cellular defenses against ROS include low-molecular antioxidants such as glutathione, thioredoxin, ascorbate, and related antioxidant enzymes like GPx, GST, catalase, and superoxide dismutase [35]. Lu et al. discovered that dehydro-PA was generated from metabolic activation of retronecine-type PA by the cocultured HepaRG hepatocytes, then triggered glutathione (GSH) depletion, increased intracellular ROS, and formed pyrrole-protein adducts [9]. The accumulation of ROS exceeds the mitochondria threshold, which will cause mitochondria damage to induce cell apoptosis, which may be one of PA cytotoxicity mechanisms. Endoplasmic reticulum dysfunction is also accompanied by a decrease in intracellular calcium ion concentration, which because of the endoplasmic reticulum acts as a cell's calcium pool. When the endoplasmic reticulum function was impaired, such as endoplasmic reticulum stress, then the calcium homeostasis in the cell will be destroyed. Previous literature reports that alkaloids can cause acute liver injury. This study found that in the HepaRG cell model, PAs caused neutral lipid metabolism disorders in a dose-dependent manner, which may be related to the acute liver injury caused by alkaloids [29]. Based on cytotoxicity and HCS assays, the following two concentrations were chosen for further analyses in HepaRG cells: 50 *μ*M (none to slightly cytotoxic) and 200 *μ*M (cytotoxic).

### 3.4. Cell Cycle Assay

**Figure 4:**
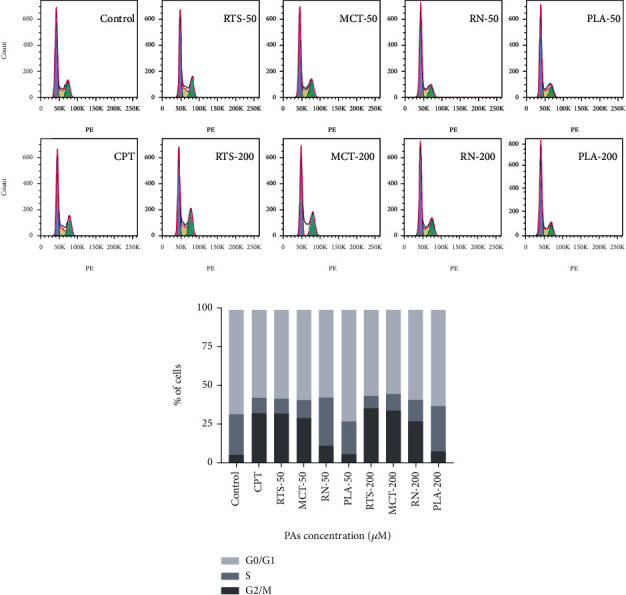
Effect of 4 PAs on the cell cycle in HepaRG cells (a, b). Distribution of cell cycle for HepaRG cells after treated with different concentrations (0, 50, and 200 *μ*M) of 4 PAs for 24 h. 10 *μ*M CPT was used as a positive control.

Apoptosis, or programmed cell death, is a complicated process that involves multiple genes. Interestingly, apoptosis could be induced during cell cycle arrest [36]. To explore whether PA-induced apoptosis was associated with cell cycle arrest, we detected the cell cycle distribution of HepaRG cells using flow cytometry to analyze cellular DNA content. As shown in Figure 4, after 24 h 4 PA treatment, there was an increase in the percentage of HepaRG cells in the G2/M phase versus control (*p* < 0.05). This phenomenon was undeniable in the RTS and MCT treatment groups. The rate of G2/M phase cells in the 200 *μ*M RTS and MCT groups was 35.40% and 33.80%, respectively (*p* < 0.01). This result is consistent with previous reports; monocrotaline treatment induced G2 phase arrest in confluent bovine pulmonary artery endothelial cells (BPAEC) [35]. Wilson et al. conclude that human pulmonary artery endothelial cells (HPAEC) treated with low concentrations of MCT develop G2 arrest in association with persistent cyclin B1 expression, failure to activate cdc2 completely, and continued DNA synthesis through a pathway that is unrelated to altered expression of p53 [37]. In contrast, there was no significant difference between the PLA treatment group and the control group.

### 3.5. Mitochondrial Membrane Potential JC-1 Assay

**Figure 5:**
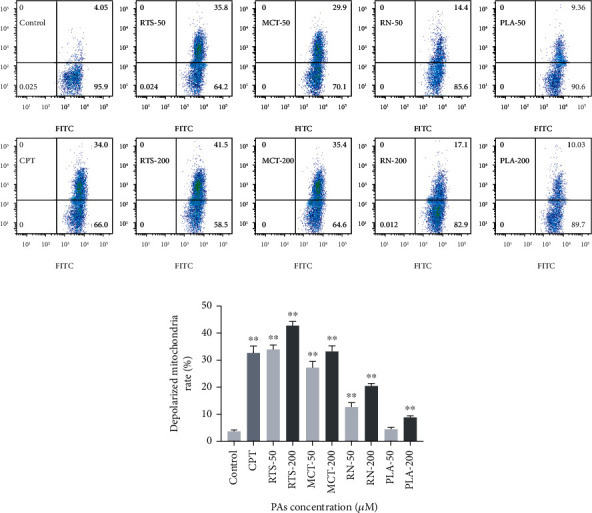
Effect of 4 PAs on the mitochondrial membrane potential of HepaRG cells (a, b). Distribution of mitochondrial membrane potential for HepaRG cells after treatment with different concentrations (0, 50, and 200 *μ*M) of 4 PAs for 24 h. 10 *μ*M CPT was used as a positive control. ^∗∗^*p* < 0.05 indicates a significant difference versus the control group.

The cells were incubated with 4 PAs for 24 hours and then stained with JC-1 to detect the mitochondrial membrane potential by flow cytometry. As shown Figure 5, CPT was used as a positive control, and its positive rate of mitochondrial depolarization was 32.7%. Compared with the control group (3.5%), the percentages of depolarized mitochondria in the RTS, MCT, and RN (50 *μ*M) treatment groups were significantly higher, 33.9%, 27.4%, and 12.7%, respectively, and with increasing concentrations. The positive rate also showed an upward trend. When the concentration was 200 *μ*M, the positive rates were 42.8%, 33.3%, and 20.6%, respectively. It is worth noting that, unlike other alkaloids, the 50 *μ*M PLA treatment group did not cause a significant increase in the positive rate of mitochondrial depolarization. Consistent with the expected results, the cells treated with RTS, MCT, and RN caused a significant loss of mitochondrial membrane potential, which may be caused by oxidative stress. Oxidative stress occurs when ROS production exceeds its depletion by antioxidant compounds or enzymes [38]. Cellular excessive ROS could react with most cellular macromolecules, such as enzymes, DNA, and protein, and the potential of the inner and outer membrane of the mitochondria will change, which will cause the depolarization of the mitochondria, furthermore, resulting in disruption of mitochondrial [39]. Interestingly, compared to RTS, MCT, and RN, PLA has no significant depolarization of mitochondria. Because retronecine-type PAs will be metabolically activated when exposed to the 1,2-unsaturated structure of the cell to generate dehydrogenated PAs, this process consumes GSH and causes ROS accumulation, while PLA is a saturated structure.

### 3.6. Cellular Apoptosis Assay

**Figure 6:**
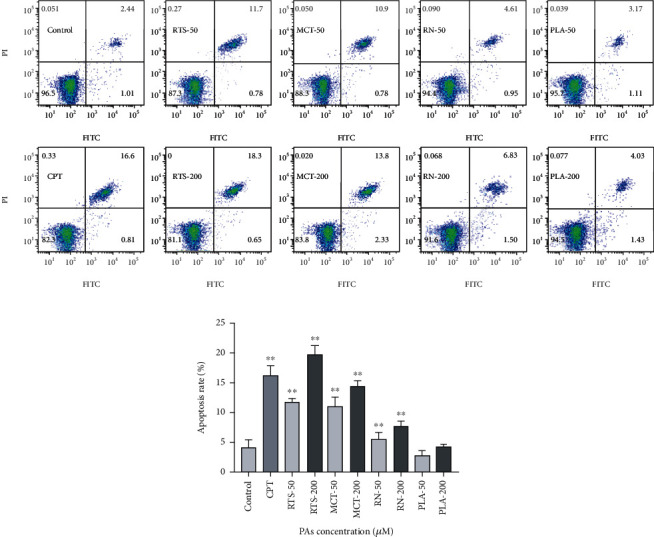
Effect of 4 PAs on the apoptosis of HepaRG cells (a, b). Distribution of apoptosis in HepaRG cells after treated with different concentrations (0, 50, and 200 *μ*M) of 4 PAs for 24 h. 10 *μ*M CPT was used as a positive control. ^∗∗^*p* < 0.05 indicates a significant difference versus the control group.

Then, we tested the cell apoptosis rate after PA treatment. The HepaRG cells were stained with Annexin-V, and apoptosis was detected by flow cytometry. The results are similar to the effects of MMP detection. Compared with the control group, except for the PLA (2.87-4.17%), the apoptosis rate of RTS (11.79-19.69%), MCT (10.92-14.37%), and RN (4.11-7.72%) was significantly increased (*p* < 0.05). It also showed a significant dose-dependent relationship (Figure 6). This result is consistent with the work of mitochondrial depolarization, suggesting that PAs may induce apoptosis through the mitochondrial pathway [24].

The previous literature in animals showed that platynecine type did not cause significant hepatic or renal damage in mice, rats, or guinea pigs treated with acute sublethal dose [29]. *In vitro* studies in rat liver presence, S9 microsomes, or human liver microsomes demonstrated that platynecine type does not undergo metabolic activation to form a reactive pyrrolic ester that can bind macromolecules like 1,2-unsaturated PAs [9]. These reactive pyrrolic esters provide a mechanism-based biomarker to assess PA toxicity [40]. Thus, platynecine type was regarded as nontoxic. However, oxidation of the saturated necine base forms a minor epoxide containing metabolite, except to a major nonreactive and water-soluble dehydroplatyphylline carboxylic acid metabolite [40]. The epoxide containing metabolite of PLA may be the activity indicative of the formation of a genotoxic metabolite [31]. Combined with our previous in silico prediction results and *in vitro* results, we speculate that PLA is not entirely nontoxic.

## 4. Conclusion

In conclusion, the present study, ADMET and TOPKAT analyses, in silico prediction, facilitated an understanding of the metabolic efficiency and possible toxicity of the different types of PAs. *In vitro* testing proved structure-dependent cytotoxicity and apoptosis after PA exposure, suggesting that the prediction of in silico has a certain reference value for compound toxicity. 12-membered cyclic diester seems to be a more potent apoptosis inducer than other PAs. In addition, we speculate that platyphylline is not entirely nontoxic. Although further consideration of biokinetics will be needed to develop a robust understanding of the relative potency for a realistic risk assessment of PAs mixtures, these data facilitate understanding their cytotoxic and apoptosis.

## Figures and Tables

**Table 1:** Effects of adsorption, distribution, metabolism, excretion, and toxicity (ADMET) prediction on 40 PAs.

Type	Compounds	AS	BBB	CYP2D6	HT	HIA	PPB
Retronecine type	Retrorsine^∗^	3	3	0	1	0	0
	Clivorine	3	3	0	1	0	0
	Riddelliine	3	3	0	1	0	0
	Senecionine	3	3	0	1	0	0
	Usaramine	3	3	0	1	0	0
	Jacobine	3	3	0	1	0	0
	Monocrotaline^∗^	4	3	0	1	0	0
	Seneciphylline	3	3	0	1	0	0
	Integerrimine	3	3	0	1	0	0
	Senecivernine	3	3	0	1	0	0
	Jacoline	4	4	0	1	0	0
	Trichodesmine	3	3	0	1	0	0
	Fulvine	3	3	0	1	0	0
	Angularine	3	3	0	1	0	0
	Crotananine	3	3	0	1	0	0
	7-Acetylintermedine	4	3	0	1	0	0
	7-Acetyllycopsamine	4	3	0	1	0	0
	Echimidine	4	4	0	1	0	0
	Echiumine	3	3	0	1	0	0
	Lycopsamine	4	3	0	1	0	0
	Intermedine	4	3	0	1	0	0
	Indicine	4	3	0	1	0	0
	Retronecine^∗^	5	3	0	1	1	0
	Lasiocarpine	3	3	0	1	0	0
	Heliosupine	4	4	0	1	0	0
	Heleurine	3	3	0	1	0	0
	Supinine	4	3	0	1	0	0
	Callimorphine	4	3	0	1	0	0
	Heliotrine	4	3	0	1	0	0
	Echinatine	4	3	0	1	0	0
	Rinderine	4	3	0	1	0	0

Platynecine type	Platyphylline^∗^	3	3	0	1	0	1
	Trachelanthamine	4	3	0	0	0	0
	Heliocoromandaline	4	3	0	0	0	0
	Heliocurassavicine	4	3	0	0	0	0

Otonecine type	Acetylanonamine	3	4	0	1	0	0
	Senkirkine	3	3	0	1	0	0
	Otosenine	3	4	0	1	0	0
	Petasitenine	3	3	0	1	0	0
	Otonecine	5	3	0	1	0	0

^∗^ *In vitro* tests compound. AS: aqueous solubility—0: extremely low; 1: very low, but possible; 2: low; 3: good; 4: very good; 5: extremely good. BBB: blood–brain barrie—0: very high penetrant; 1: high; 2: medium; 3: low; 4: undefined. CYP2D6: cytochrome P450 2D6—0: noninhibitor; 1: inhibitor. HT: hepatotoxicity—0: nontoxic; 1: toxic. HIA: human intestinal absorption—0: good; 1: moderate; 2: poor; 3: very poor. PPB: protein plasma binding—0: absorbent weakly; 1: absorbent highly.

**Table 2:** Effects of toxicity predictive test on 40 PAs.

Type	Compounds	Mouse NTP^a^		Rat NTP^a^		Ames^b^	DTP^c^	LD_50_ (g/kg)	LOAEL (g/kg)
		F	M	F	M				
Retronecine type	Retrorsine^∗^	1	1	1	1	0	1	0.320	0.001
	Clivorine	1	1	1	1	0	1	0.386	0.002
	Riddelliine	1	1	0	0	0	1	0.616	0.015
	Senecionine	0	0	0	1	0	1	0.127	0.001
	Usaramine	0	1	1	1	0	1	0.264	0.002
	Jacobine	1	1	1	1	0	1	0.461	0.003
	Monocrotaline^∗^	1	1	1	1	0	1	0.731	0.002
	Seneciphylline	0	1	1	1	0	1	0.264	0.002
	Integerrimine	1	1	1	1	0	1	0.254	0.002
	Senecivernine	0	1	1	1	0	1	0.592	0.004
	Jacoline	1	1	1	1	1	1	0.230	0.001
	Trichodesmine	0	1	1	1	0	1	0.324	0.004
	Fulvine	1	1	1	1	1	1	0.369	0.002
	Angularine	0	1	0	0	0	1	0.559	0.009
	Crotananine	0	1	1	1	0	1	0.592	0.004
	7-Acetylintermedine	0	1	0	0	0	1	0.559	0.009
	7-Acetyllycopsamine	0	1	0	1	0	1	0.356	0.003
	Echimidine	1	1	0	0	0	1	0.616	0.015
	Echiumine	1	1	1	1	0	1	0.122	0.001
	Lycopsamine	1	1	1	1	0	1	0.239	0.001
	Intermedine	0	1	1	1	0	1	0.264	0.002
	Indicine	0	1	1	1	0	1	0.264	0.002
	Retronecine^∗^	1	1	1	1	0	1	0.242	0.001
	Lasiocarpine	1	1	1	1	1	1	0.555	0.001
	Heliosupine	0	1	1	1	1	1	0.708	0.002
	Heleurine	1	1	0	0	0	1	0.616	0.015
	Supinine	0	1	1	1	0	1	0.215	0.001
	Callimorphine	0	1	0	0	0	1	0.559	0.009
	Heliotrine	0	0	0	1	0	1	0.056	0.001
	Echinatine	1	1	0	1	0	1	0.250	0.003
	Rinderine	1	1	1	1	0	1	0.486	0.001

Platynecine type	Platyphylline^∗^	1	1	1	1	0	1	0.443	0.002
	Trachelanthamine	1	1	1	1	1	1	0.391	0.001
	Heliocoromandaline	1	1	1	1	0	1	0.246	0.004
	Heliocurassavicine	1	1	1	1	0	1	0.404	0.001

Otonecine type	Acetylanonamine	1	1	1	1	1	1	0.230	0.001
	Senkirkine	1	1	1	1	1	1	0.275	0.001
	Otosenine	0	1	1	1	1	1	0.106	0.001
	Petasitenine	0	1	1	1	0	1	0.264	0.002
	Otonecine	1	1	1	1	0	1	0.467	0.001

^∗^ *In vitro* test compound. NTP: U.S. National Toxicology Program rodent carcinogenicity; F: female; M: male; DTP: developmental toxicity potential; LD_50_: rat oral LD_50_ (g/kg); LOAEL: rat chronic oral LOAEL (g/kg). *a* < 0.3 (noncarcinogen); >0.8 (carcinogen). *b* < 0.3 (nonmutagen); >0.8 (mutagen). *c* < 0.3 (nontoxic); >0.8 (toxic).

## Data Availability

All data generated or analyzed during this study are included in this article.
